# How does physical activity alleviate nurse job burnout? The important role of recovery experiences

**DOI:** 10.3389/fpubh.2025.1658020

**Published:** 2025-09-11

**Authors:** Xingyi Li, Tianci Zhang, Guobin Zhao, Jie Li, Changzhou Chen

**Affiliations:** ^1^Department of Sport and Health, Shinhan University, Uijeongbu-si, Republic of Korea; ^2^Department of Sports Integration, Namseoul University, Cheonan-si, Republic of Korea; ^3^School of Nursing, Anqing Medical College, Anqing, China; ^4^School of Physical Education, Shanghai University of Sport, Shanghai, China

**Keywords:** physical activity, job burnout, recovery experience, recovery activity, nurse

## Abstract

**Background:**

In high-intensity work environments, nurses are significantly affected by job burnout. This study aims to explore the relationship between physical activity and nurses’ job burnout, while examining the mediating role of recovery experiences.

**Method:**

Convenience sampling method was employed to recruit 912 nurses. Data were collected using the Physical Activity Rating Scale, Recovery Experience Questionnaire, and Job Burnout Inventory. Empirical analyses were conducted, and the bootstrapping method was applied to test the mediating effects of recovery experiences.

**Results:**

Physical activity significantly and negatively related nurses’ job burnout (*β* = −0.554, *p* < 0.01). The mediating effects of psychological detachment [95% CI (−0.217 ~ −0.168)], relaxation experience [95% CI (−0.081 ~ −0.045)], and mastery experience [95% CI (−0.228 ~ −0.177)] in the relationship between physical activity and job burnout were confirmed. However, the mediating effect of control experience [95% CI (−0.020 ~ 0.001)] was not statistically significant.

**Conclusion:**

This study identifies a significant negative association between physical activity and job burnout among nurses, and highlights the multidimensional contributions of recovery experiences to this association. The findings provide empirical evidence that may inform strategies for enhancing nurses’ occupational health.

## 1 Introduction

Nurses, as indispensable professionals within healthcare teams (1), are consistently exposed to high-intensity and high-demand work environments, rendering them particularly vulnerable to job burnout (2). Research indicates that job burnout has become prevalent among nurses globally, with significant rates observed across multiple countries. For instance, the prevalence of job burnout among nurses in Europe and the United States is 20.90 and 16.60–30% (3, 4), while rates in Japan and China reach 56 and 35.50–50%, respectively (5, 6). Job burnout is defined as a state of physical and mental exhaustion resulting from prolonged engagement in high-stress, high-workload occupations (7). Its emergence stems from the depletion of individuals’ physiological and psychological resources, leaving them inadequately equipped to meet job demands (8). At the same time, the shortage of nurses is becoming more and more serious globally, and according to the World Health Organization, it is projected that 9 million new nurses will be needed globally by 2030 to meet the healthcare demand (9). In addition to the existing shortage of nursing human resources, recent years have witnessed a substantial number of nurses voluntarily resigning, with some even leaving the nursing profession entirely, further exacerbating the problem (10). Evidence suggests that job burnout is a significant predictor of nurses’ turnover intention (11). At the same time, nursing work imposes exceptionally high psychological and professional demands: nurses are required not only to communicate effectively with patients in high-pressure environments (12), but also to make accurate judgments and decisions rapidly in emergency situations. Such sustained, high-intensity workloads can easily lead to severe psychological stress, thereby further triggering burnout and turnover behaviors (13). Nurse burnout has numerous adverse effects on both individuals and patients. It can lead to mental health problems such as depression and anxiety among nurses (14, 15), while also undermining the quality of patient care, increasing the likelihood of medical errors, and jeopardizing patient safety (16). Therefore, against this background, how to alleviate nurse burnout has become an important issue that needs to be urgently addressed.

Resource conservation theory is a key framework for understanding work recovery. Resource conservation theory recognizes that individuals have a tendency to strive to acquire and conserve resources because they are essential to their adaptation to their environment and pursuit of their goals (17). At the same time, resource conservation theory further states that all activities that contribute to the acquisition of new resources (e.g., physical activity, socialization, etc.) are beneficial to the realization of recovery (18). Physical activity, as a recovery activity that can effectively improve the psychological health of individuals, can not only effectively alleviate the negative emotions of individuals, but also significantly enhance their sense of well-being (19). Work recovery is often divided into two categories, namely recovery activities and recovery experiences. Recovery activities mainly focus on the specific behaviors individuals engage in during the recovery process, whereas recovery experiences refer to the psychological experiences during the process (20). Empirical studies have shown that recovery activities and recovery experiences have different effects on individuals’ work status, but they are interrelated (21). Among them, recovery experience can help individuals effectively recover physical and psychological resources, thus alleviating burnout (22).

Existing studies on nurse burnout have examined the influencing factors of nurse burnout at two levels: work environment and personal characteristics. The work environment level involves factors such as workload (23), staffing (24) and work patterns (25), while the individual level includes factors such as job control (26), values (27) and sense of fairness (28) among other factors. In addition, it has been shown that physical activity plays a positive role in alleviating nurse burnout (29). However, although previous studies have revealed the multidimensional causes of burnout and verified the role of physical activity in alleviating burnout, the following deficiencies still exist. First, although some studies have paid attention to the association between physical activity and nurse burnout, the theoretical interpretation of the findings in existing studies is obviously insufficient, failing to fully combine the existing theoretical frameworks to analyze the findings in depth, thus limiting the in-depth understanding of the phenomenon. Secondly, the existing studies mainly focus on the direct effect of physical activity on burnout, but the internal logic of its mechanism has not been explored in depth, especially the key question of why physical activity can effectively alleviate burnout needs to be further explored.

In summary, in order to fill this research gap, the present study, based on the analytical framework of resource conservation theory, delves into the relationship between physical activity and nurse burnout from the perspective of work recovery, and further introduces the recovery experience as a mediating variable in order to reveal the intrinsic mechanism of physical activity in alleviating burnout. Through this theoretical integration framework, this study not only expands the application of resource preservation theory in the study of burnout, but also provides more practical intervention ideas for improving nurse burnout.

## 2 Theoretical framework

### 2.1 Physical activity and job burnout

Research on work recovery has pointed out that physical activity has received extensive academic attention as a recovery activity (30). As one of the favorite leisure activities of workers during non-work hours, the important role of physical activity for physical and mental health is supported by numerous studies (31). Findings in sport psychology have demonstrated that engaging in physical activity enhances various physiological and psychological indicators associated with antidepressant effects, including elevated levels of endorphins, epinephrine, and other neurochemicals (32). Empirical studies in the field of occupational health have also examined the restorative effects of physical activity in terms of physical and mental health, with researchers finding that regular participation in physical activity played a positive role in alleviating workers’ burnout (33). The higher the level of physical vigor (34), the higher the individual’s positive mood (35), and the higher the satisfaction and happiness with work and family (36). Based on the theory of resource conservation, individuals deplete a large amount of physical and psychological resources due to the demands of their work, and the recovery of non-work time signifies the end of resource depletion and the beginning of resource replenishment (37). Hu’s study also supports the above analysis, as nurses who participated in physical activity on a daily basis were less likely to experience burnout (38). Regular participation in physical activity showed a strong correlation with a decrease in nurse burnout (39). Therefore, combining the results of research in several fields mentioned above, the present study concludes that physical activity can serve as a highly effective recovery activity that can provide individuals with adequate resource replenishment to alleviate burnout. Accordingly, Hypothesis 1 is proposed:

*H1*: Physical activity can be significantly and negatively related to the level of nurses’ job burnout.

### 2.2 Mediating role of the recovery experiences

Recovery experiences refer to the psychological experiences during the work recovery process and constitute the process mechanism through which recovery effects occur (40). Specifically, recovery experiences are the states of recovery, relaxation, and replenishment of energy that individuals experience after work ends, akin to the feeling of recharging, which helps alleviate fatigue and stress incurred during work (41). Recovery experiences encompass four dimensions (40), psychological disengagement, relaxation experience, mastery experience, and control experience. Psychological disengagement refers to the mental state in which employees detach from work, meaning they cease to focus on or think about work-related matters during non-working hours. Relaxation experience describes the psychological state in which employees achieve a sense of relaxation through various activities during their rest periods. This experience enables individuals to acquire positive emotional perception, reduce stress levels, and temporarily escape from work - related troubles. Mastery experience refers to the experience in which workers acquire the experience and ability to overcome difficulties and challenges by learning other knowledge unrelated to work and mastering new skills. Control experience is the psychological experience in which workers autonomously choose, plan, and decide when and how to do something or engage in an activity during non - working time, thereby enabling individuals to perceive a sense of autonomy or dominance. Studies have shown that most recovery activities are positively correlated with recovery experiences (30). Of these, non-competitive physical activity demonstrated a strong correlation. On the one hand, when individuals participate in physical activities, such as running, yoga, swimming, etc., their blood circulation is promoted, and the nerves and muscles that are tense during work are relaxed and soothed, allowing the body to relax and recover. At the same time, physical activities stimulate the secretion of endorphins and dopamine, causing individuals to experience pleasure (35). Nursing work is characterized by high intensity and high emotional investment (42). The pleasure generated by endorphins and dopamine can help nurses replenish emotional resources (43). Therefore, by participating in physical activities, individuals can not only have the opportunity to obtain new resources such as a sense of belonging, a sense of achievement, and self - efficacy but also will not over - consume their existing resources (40). This indicates that participating in physical activities during non - working time is a process that can quickly achieve resource reconstruction and obtain new resources. The longer the time individuals invest in physical activities, the higher the level of recovery experiences they obtain.

The resource replenishment and restructuring brought about by recovery activities are achieved by shaping the recovery experiences perceived by individuals, thereby affecting work performance (36). The conservation of resources theory posits that work leads to the loss of individual resources, and the substantial loss of these resources can result in burnout. Consequently, individuals tend to prevent resource loss, conserve existing resources, and acquire new ones (44). According to this theory, individuals can achieve psychological detachment from work through participation in physical activity, thereby preventing resource loss and facilitating resource conservation and acquisition through recovery experiences. Numerous empirical studies have demonstrated a close intrinsic relationship between recovery experiences and burnout. Sonnentag and colleagues found a negative correlation between the psychological detachment dimension of recovery experiences and the emotional exhaustion dimension of burnout (40). Elset and others discovered a negative correlation between the mastery experience dimension of recovery experiences and the emotional exhaustion dimension of burnout (45). Poulsen and colleagues also found a negative correlation between recovery experiences and burnout (46). In summary, recovery experiences enable individuals to recharge during non-work time, enhancing their energy and motivation, reducing stress, and generating positive emotions (47). This, in turn, helps maintain a good physical and mental state during work, ultimately achieving the goal of alleviating burnout. Accordingly, this study suggests that recovery experiences may mediate the relationship between physical activity and nurses’ job burnout, and Hypothesis 2 is hereby presented: Recovery experience mediates the relationship between physical activity and nurse burnout.

*H2*: Recovery experience mediates the relationship between physical activity and nurse burnout.

*H2a*: Psychological disengagement mediates the relationship between physical activity and nurse burnout.

*H2b*: Relaxation experience mediates between physical activity and nurse burnout.

*H2c*: Mastery experience mediates between physical activity and nurse burnout.

*H2d*: Control experience mediates between physical activity and nurse burnout.

## 3 The present study

**Figure 1:**
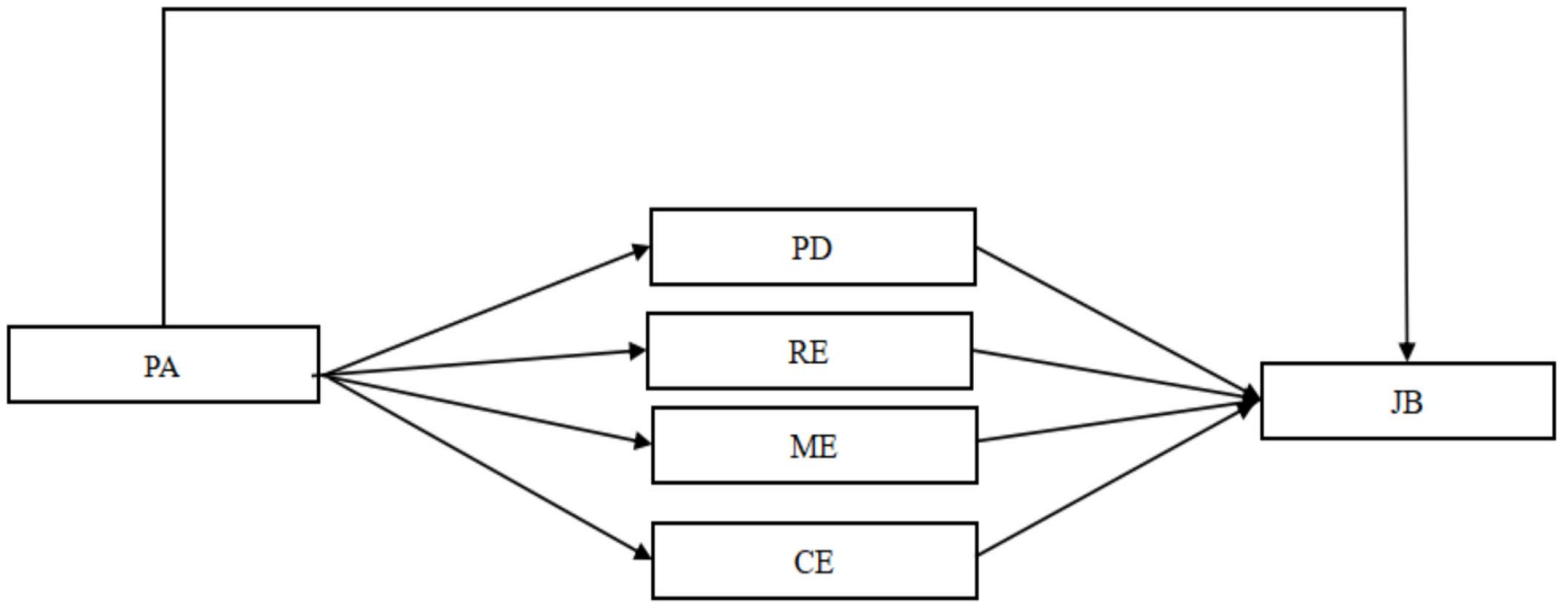
Concept model. PA, physical activity; JB, job burnout; PD, psychological disengagement; RE, relaxing experience; ME, mastery experience; CE, control experience.

In conclusion, this study has delved into the relationship between physical activity and nurses’ job burnout in depth, and by introducing the concept of recovery experiences into the discussion, it has revealed how physical activity influences nurses’ job burnout. As shown in Figure 1, we have constructed an overall conceptual model and proposed the following hypotheses:

*H1*: Physical activity can be significantly and negatively related to the level of nurses’ job burnout.

*H2*: Recovery experience mediates the relationship between physical activity and nurse burnout.

*H2a*: Psychological disengagement mediates the relationship between physical activity and nurse burnout.

*H2b*: Relaxation experience mediates between physical activity and nurse burnout.

*H2c*: Mastery experience mediates between physical activity and nurse burnout.

*H2d*: Control experience mediates between physical activity and nurse burnout.

## 4 Methods

### 4.1 Participants

This study adopted a convenience sampling method and conducted a questionnaire survey in several general hospitals located in Anhui, Shandong, and Shanghai, China. Through the recommendations of existing participants, it was more convenient to reach more subjects. Researchers fully communicated with the subjects, explained the value and significance of the research to them, obtained their permission to participate, and invited them to take part in our questionnaire survey. At the same time, prior to the survey, an ‘informed consent form’ was distributed and written consent was obtained from each subject. As show in Table 1, total of 946 questionnaires were distributed. Excluding invalid questionnaires caused by completely inconsistent answers, a total of 912 valid questionnaires were retained, resulting in an effective response rate of 96.41%. Meanwhile, appropriate incentive measures were implemented to encourage participants’ cooperation, and each participant received 20 yuan as compensation upon completion of the survey. Among the respondents, there were 793 females (87.0%) and 119 males (13.0%). Regarding educational background, 398 participants (43.6%) had education below the undergraduate level, 417 (45.7%) held an undergraduate degree, and 97 (10.6%) had a master’s degree or above. In terms of marital status, 442 participants (48.5%) were married and 470 (51.5%) were unmarried. As for departmental distribution, 212 participants (23.3%) worked in the emergency department, 246 (27.0%) in the internal medicine department, 153 (16.8%) in the surgical department, 142 (15.6%) in the gynecology and obstetrics department, and 159 (17.4%) in the pediatrics department. This study was conducted in strict accordance with the Declaration of Helsinki and was approved by the Human Ethics Committee of Anqing Medical College (Approval No. 2025–04-001). Informed consent was obtained from all participants.

**Table 1:** Demographic characteristics of the participants.

Variable	Category	*N*	Percentage (%)
Gender	Female	793	87
	Male	119	13
Education background	Below undergraduate	398	43.6
	Undergraduate	417	45.7
	Master’s or above	97	10.6
Department	Emergency	212	23.3
	Internal medicine	246	27
	Surgery	153	16.8
	Gynecology and obstetrics	142	15.6
	Pediatrics	159	17.4
Marital status	Married	176	90.7
	Unmarried		

### 4.2 Instruments

#### 4.2.1 Demographic variables

Demographic variables collected included gender, age, marital status, and education level. Work-related variables included department, job title, and working years. These variables were self-reported via single-choice questions in the questionnaire.

#### 4.2.2 Physical activity

Physical activity was assessed using the Physical Activity Rating Scale originally developed by Japanese scholar Kouji Hashimoto (48) and subsequently revised by Chinese scholar Deqing Liang to better suit the sociocultural context of China (49). The scale evaluates physical activity over the past month across three behavioral dimensions: exercise intensity, exercise duration, and exercise frequency. Each dimension is measured on a multiple-response ordinal scale with five ordered categories reflecting behavioral levels. Specifically, exercise frequency ranges from “less than once per month” to “almost every day”; exercise duration ranges from “under 10 min” to “over 60 min” per session; and exercise intensity ranges from subjectively perceived “light activity” to “high-intensity sustained exercise.” Exercise volume is then calculated using the formula: exercise volume = exercise frequency × (exercise duration − 1) × exercise intensity, yielding scores from 0 to 100, with higher scores indicating greater overall physical activity. The applicability of this revised scale among Chinese university students has been widely validated (50).

#### 4.2.3 Recovery experiences

The Recovery Experience Questionnaire developed by Sonnentag et al. (40) was used to measure recovery experience. The scale comprises four dimensions—mastery experience, psychological detachment, control experience, and relaxation experience—with a total of 16 items. Each item is rated on a 5-point scale ranging from 1 (“not at all”) to 5 (“fully”), based on the respondent’s actual non-work experience, with higher scores indicating higher levels of recovery experience. In this study, the scale was translated into Chinese following a standardized forward–backward translation procedure. First, two bilingual psychology researchers independently translated the original English version into Chinese. Another bilingual researcher then back-translated the preliminary Chinese version into English. The research team compared the back-translated version with the original scale, discussed semantic discrepancies, and reached consensus to ensure that the translation was both faithful to the original meaning and culturally appropriate in Chinese. The applicability of this scale in Chinese nursing populations has been widely validated, with consistently high reliability and validity (51, 52). In the present study, the overall Cronbach’s alpha coefficient for the scale was 0.865. For the four dimensions, the Cronbach’s alpha values were as follows: mastery experience (*α* = 0.876), psychological detachment (α = 0.826), control experience (α = 0.791), and relaxation experience (α = 0.826). Confirmatory factor analysis yielded the following fit indices: χ^2^/df = 2.824, NFI = 0.982, TLI = 0.953, CFI = 0.976, and RMSEA = 0.046. All indices met conventional standards (53), indicating that the scale demonstrated good reliability and validity in this study.

#### 4.2.4 Job burnout

Job burnout was assessed using the Job Burnout Scale developed by Li et al. (54) specifically designed to reflect the occupational characteristics of Chinese nurses. The scale consists of three dimensions: emotional exhaustion (e.g., “Working with patients is a burden to me”), depersonalization (e.g., “I do not attach much importance to the various requests made by patients”), and personal accomplishment (e.g., “Engaging in nursing work allows me to realize my self-worth”), comprising a total of 22 items. Responses are rated on a 7-point Likert scale ranging from 1 (“never”) to 7 (“every day”). In the present study, the overall Cronbach’s alpha coefficient for the scale was 0.952. For the three dimensions, the Cronbach’s alpha values were as follows: emotional exhaustion (*α* = 0.929), depersonalization (α = 0.897), and personal accomplishment (*α* = 0.928). Confirmatory factor analysis yielded the following fit indices: χ^2^/df = 2.624, NFI = 0.948, TLI = 0.949, CFI = 0.954, and RMSEA = 0.043. All indices met conventional criteria (53), indicating that the scale demonstrated good reliability and validity in this study.

### 4.3 Data analysis

This study analyzes the data through the following steps. Firstly, Cronbach’s α coefficient and confirmatory factor analysis (CFA) were used to test the reliability and validity of the scales, respectively. Secondly, to prevent serious common method bias, Harman’s single factor test was applied to evaluate potential common method variance (55). Thirdly, a partial correlation analysis was conducted to examine the associations among the variables in the conceptual model, controlling for gender, education level, marital status, and department. Statistical significance was assessed using two-tailed tests. Finally, in the hypothesis-testing stage, mediation effects were examined using the PROCESS macro developed by Hayes (56). Specifically, the bootstrap method was applied with 5,000 resamples to generate the mediation effect distribution, enhancing the robustness of estimation. In each resample, the direct and indirect path coefficients between variables were calculated, and the significance of the mediation effect was judged based on the 95% confidence interval (CI), if the interval did not contain zero, the mediation effect was considered significant (57). This procedure effectively controls sampling error and improves the reliability of statistical inference.

## 5 Result

### 5.1 Common method test

In order to comprehensively assess common method variance in this study, Harman’s single-factor test was conducted. The test results show that the maximum one-factor explained variance is 36.90%, which is lower than the critical value of 40%, which indicates that there is no single dominant factor in the data, and preliminarily verifies that there is no serious common method bias problem in this study.

### 5.2 Correlation analysis

As shown in Table 2, the means, standard deviations and correlation coefficients of the variables are demonstrated. The results indicated that physical activity was significantly and negatively associated with job burnout (*r* = −0.555, *p* < 0.01), while it was significantly and positively associated with psychological disengagement (*r* = 0.463, *p* < 0.01), relaxing experience (*r* = 0.259, *p* < 0.01), mastery experience (*r* = 0.438, *p* < 0.01), and control experience (*r* = 0.209, *p* < 0.01). Job burnout was significantly and negatively associated with psychological disengagement (*r* = −0.582, *p* < 0.01), relaxing experience (*r* = −0.367, *p* < 0.01), mastery experience (*r* = −0.616, *p* < 0.01), and control experience (*r* = −0.155, *p* < 0.01). Psychological disengagement was significantly and positively associated with relaxing experience (*r* = 0.323, *p* < 0.01), mastery experience (*r* = 0.490, *p* < 0.01), and control experience (*r* = 0.280, *p* < 0.01). Relaxing experience was significantly and positively associated with mastery experience (*r* = 0.386, *p* < 0.01) and control experience (*r* = 0.202, *p* < 0.01). Finally, mastery experience was also significantly and positively associated with control experience (*r* = 0.328, *p* < 0.01).

**Table 2:** Correlation analysis.

Variable	Mean	SD	1	2	3	4	5	6
1. PA	21.38	21.92	1					
2. JB	5.45	0.81	−0.555**	1				
3. PD	5.43	1.03	0.463**	−0.582**	1			
4. RE	5.48	0.80	0.259**	−0.367**	0.323**	1		
5. ME	5.40	0.98	0.438**	−0.616**	0.490**	0.386**	1	
6. CE	5.50	0.73	0.209**	−0.155**	0.280**	0.202**	0.328**	1

G, gender; EA, education attainment; MS, marital status; D, department; PA, physical activity; JB, job burnout; PD, psychological disengagement; RE, relaxing experience; ME, mastery experience; CE, control experience; *p < 0.05; **p < 0.01.

### 5.3 Mediating effects test

In this study, the mediating effect of recovery experience was tested by process plug-in in SPSS 26.0 and by applying the bootstrap method with 5,000 repetitive samples. The results of the test are shown below.

As shown in Tables 3, 4, the mediating effect of psychological disengagement was examined after controlling for gender, education, marital status, and department. The direct effect of physical activity on nurse burnout was first tested without including the mediating variable, and the results showed that physical activity was significantly and negatively related to burnout (*β* = −0.554, *p* < 0.01). After adding psychological disengagement into the model, physical activity was significantly and positively related to psychological disengagement (*β* = 0.463, *p* < 0.01), and psychological disengagement was significantly and negatively related to job burnout (*β* = −0.412, *p* < 0.01). The direct effect of physical activity on burnout remained significant (*β* = −0.363, *p* < 0.01). Furthermore, the bias-corrected bootstrap method was used to test the significance of the mediating effect. The results indicated that psychological disengagement mediated the association between physical activity and job burnout (Indirect effect = −0.191, 95% BootCI = −0.217 ~ −0.168), Hypothesis H2a is verified.

**Table 3:** Mediation model test of psychological disengagement.

Variable	JB	PD	JB
G	−0.034	0.014	−0.028
EA	0.073	−0.004	0.071
MS	0.032	0.011	0.036
D	−0.016	0.006	−0.013
PA	−0.554**	0.463**	−0.363**
PD			−0.412**
R ^2^	0.312	0.215	0.445
Adjust R ^2^	0.308	0.210	0.442

G, gender; EA, education attainment; MS, marital status; D, department; PA, physical activity; JB, job burnout; PD, psychological disengagement; *p < 0.05, **p < 0.01.

**Table 4:** Test of the mediation effect of the Bootstrap method on psychological disengagement.

Path	Total effect	Indirect effect	95%CI	Direct effect	Percentage of total effect
PA → PD → JB	−0.554**	−0.191	−0.217 ~ −0.168	−0.363**	34.46%

PA, physical activity; PD, psychological disengagement; JB, job burnout. *p < 0.05; **p < 0.01.

As shown in Tables 5, 6, the mediating effect of relaxation experience was examined after controlling for gender, education, marital status, and department. After including the mediating variable in the model, physical activity was significantly and positively related to relaxation experience (*β* = 0.259, *p* < 0.01), and relaxation experience was significantly and negatively related to job burnout (β = −0.239, p < 0.01). Meanwhile, the direct effect of physical activity on burnout remained significant (*β* = −0.492, *p* < 0.01). Furthermore, the bias-corrected bootstrap method was used to test the significance of the mediating effect. The results indicated that relaxation experience mediated the association between physical activity and job burnout (Indirect effect = −0.062, 95% BootCI = −0.081 ~ −0.045), Hypothesis H2b is verified.

**Table 5:** Mediation model test of relaxation experience.

Variable	JB	RE	JB
G	−0.034	0.114	−0.007
EA	0.073	0.009	0.075
MS	0.032	0.054	0.045
D	−0.016	−0.034	−0.024
PA	−0.554**	0.259**	−0.492**
RE			−0.239**
R^2^	0.312	0.07	0.365
Adjust R^2^	0.308	0.065	0.361

G, gender; EA, education attainment; MS, marital status; D, department; PA, physical activity; JB, job burnout; RE, relaxing experience; *p < 0.05; **p < 0.01.

**Table 6:** Test of the mediation effect of the Bootstrap method on relaxation experience.

Path	Total effect	Indirect effect	95%CI	Direct effect	Percentage of total effect
PA → RE → JB	−0.554**	−0.062	−0.081 ~ −0.045	−0.492**	11.17%

PA, physical activity; JB, job burnout; RE, relaxing experience; *p < 0.05; **p < 0.01.

As shown in Tables 7, 8, the mediating effect of mastery experience was examined after controlling for gender, education, marital status, and department. After including the mediating variable in the model, physical activity was significantly and positively related to mastery experience (*β* = 0.438, *p* < 0.01), and mastery experience was significantly and negatively related to burnout (β = −0.461, *p* < 0.01). Meanwhile, the direct effect of physical activity on burnout remained significant (*β* = −0.352, p < 0.01). Furthermore, the bias-corrected bootstrap method was used to test the significance of the mediating effect. The results indicated that mastery experience mediated the association between physical activity and job burnout (Indirect effect = −0.202, 95% BootCI = −0.228 ~ −0.177), Hypothesis H2c is verified.

**Table 7:** Mediation model test of mastery experience.

Variable	JB	ME	JB
G	−0.034	0.084	0.005
EA	0.073	−0.009	0.069
MS	0.032	−0.027	0.02
D	−0.016	−0.004	−0.018
PA	−0.554**	0.438**	−0.352**
ME			−0.461**
R ^2^	0.312	0.193	0.483
Adjust R ^2^	0.308	0.189	0.480

G, gender; EA, education attainment; MS, marital status; D, department; PA, physical activity; JB, job burnout; ME, mastery experience; *p < 0.05; **p < 0.01.

**Table 8:** Test of the mediation effect of the bootstrap method on mastery experience.

Path	Total effect	Mediation effect value	95% CI	Direct effect	Percentage of total effect
PA → ME→JB	−0.554**	−0.202	−0.228 ~ −0.177	−0.352**	36.47%

PA, physical activity; JB, job burnout; ME, mastery experience; *p < 0.05; **p < 0.01.

**Figure 2:**
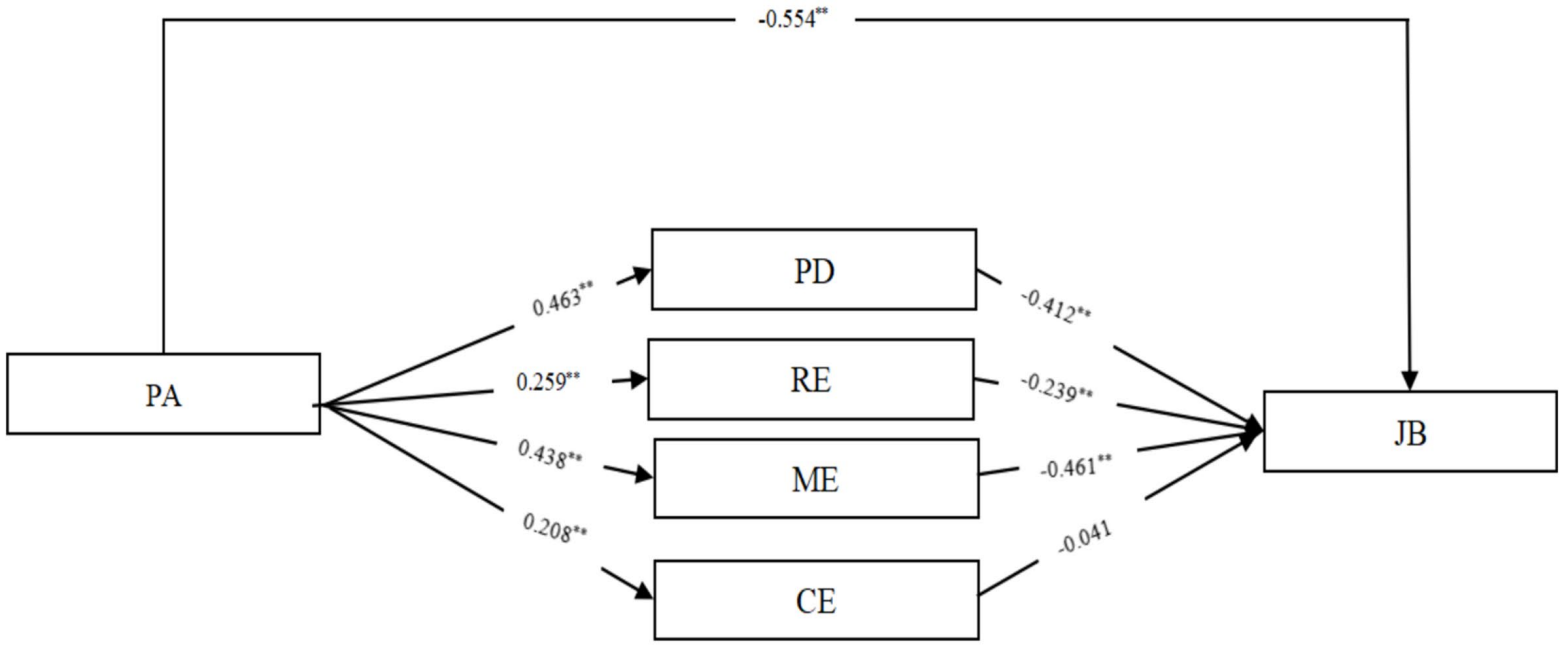
Test of the mediation effect of the recovery experience.

As shown in Tables 9, 10, the mediating effect of control experience was tested while controlling for gender, education, marital status, and department. After introducing the mediating variable mastery experience into the model, physical activity was significantly and positively related to control experience (*β* = 0.208, *p* < 0.01). However, the effect of control experience on burnout was not significant (*β* = −0.041, *p* > 0.05). Despite this, the direct effect of physical activity on burnout remained significant (*β* = 0.546, p < 0.01). The bias-corrected bootstrap method was used to test the indirect effect. The results showed that the indirect effect was negative and not significant (Indirect effect = −0.009, 95% CI = −0.020 ~ 0.001), as the confidence interval included zero. Therefore, the mediating role of control experience in the relationship between physical activity and burnout was not supported, and Hypothesis 2d was not confirmed. A summary of the mediating effect test results for recovery experience is presented in Figure 2.

**Table 9:** Mediation model test of control experience.

Variable	JB	CE	JB
G	−0.034	0.144	−0.028
EA	0.073	0.099*	0.077
MS	0.032	−0.046	0.03
D	−0.016	−0.036	−0.017
PA	−0.554**	0.208**	−0.546**
CE			−0.041
R^2^	0.312	0.05	0.314
Adjust R^2^	0.308	0.045	0.309

G, gender; EA, education attainment; MS, marital status; D, department; PA, physical activity; JB, job burnout; CE, control experience; *p < 0.05; **p < 0.01.

**Table 10:** Test of the mediation effect of the Bootstrap method on control experience.

Path	Total effect	Mediation effect value	95%CI	Direct effect	Percentage of total effect
PA → CE → JB	−0.554**	−0.009	−0.020 ~ 0.001	−0.546**	0%

PA, physical activity; JB, job burnout; CE, control experience; *p < 0.05; **p < 0.01.

## 6 Discussion

This study investigated the relationship between physical activity and nurses’ job burnout, as well as the mediating roles of different dimensions of recovery experiences. The findings revealed that physical activity is significantly and negatively related to nurses’ job burnout. Moreover, psychological disengagement, relaxation experience, and mastery experience were found to mediate the relationship between physical activity and job burnout, whereas the mediating effect of control experience was not supported.

The research results indicate that physical activity is significantly and negatively related to the level of nurses’ job burnout, which supports Hypothesis H1. This finding not only expands the related research on work recovery but also validates the applicability of the conservation of resources (COR) theory in the medical group and broadens the application scope of the theory. Nurses have long been facing huge work pressure (58) and complex doctor - patient relationships (59), which will cause a great loss to their psychological resources (60). Physical activity can alleviate the negative emotions brought by work. From the perspective of work recovery, participating in recovery activities provides workers with opportunities for resource replenishment and reconstruction (61). The resources lost by nurses due to high - intensity work requirements can be supplemented to a certain extent through participation in physical activity, thereby effectively alleviating their level of job burnout. In addition, Chokri’s research shows that physical activity has no relieving effect on the job burnout of medical staff, which is in sharp contrast to this study (62). This may be due to differences in research backgrounds. The above - mentioned research focuses on medical staff during the COVID - 19 pandemic, when the driving factors of job burnout are special, such as infection risk, tight medical resources, excessive workload, and multiple stressors coexisting such as isolation (63). This extreme environment may cause individuals’ stress thresholds to be broken through, making the recovery benefits of conventional recovery activities (such as physical activity) significantly reduced. However, this study was conducted under a non - epidemic background, where stressors may be more concentrated on conventional occupational loads, and the recovery effect of physical activity is more likely to be shown. This also implies the importance of environmental factors for the recovery of nurses’ job burnout.

The findings indicate that psychological disengagement, relaxation experience, and mastery experience significantly mediate the relationship between physical activity and nurses’ job burnout, whereas the mediating effect of control experience is not supported. Studies have shown that workers can obtain recovery experiences through participating in activities such as physical activity and recover from work stress (40). Bennett’s meta - analysis also found that different dimensions of recovery experiences can alleviate job burnout and increase the sense of energy (64). First, the mediating effect of psychological disengagement between physical activity and nurses’ job burnout is established, which verifies Hypothesis H2a. psychological disengagement can promote the recovery of physical and mental resources and reduce emotional consumption (65). A good level of psychological detachment not only allows nurses to physically escape from the work environment but also keeps them mentally detached from work. This psychological “disconnection” can completely free nurses from work - related thoughts that consume individual psychological resources, thereby reducing the damage of work stress to the body and mind and alleviating the burnout caused by occupational stress (66). Sonnentag et al. believe that individuals must make efforts to meet work requirements, and these efforts will consume individual resources and cause physiological reactions such as fatigue. Short - term rest can help individuals return to the baseline state. During rest, work requirements no longer have an impact on individuals, and work - load responses will gradually decrease and disappear (67). When participating in physical activity, individuals no longer need to engage in mental labor and consider social demands, and they will enter a “brake” mode from the high - energy - consuming work state (68). Resource consumption begins to decrease, and the activation level gradually lowers. Therefore, participating in physical activity can take nurses out of the original high - pressure work environment and enter a physical and mental state that is completely different from the occupational situation. In this process, physical movement replaces the consumption of mental labor, and the complexity of social demands is also temporarily simplified, thereby improving their level of psychological disengagement. It helps nurses face work with positive and development - promoting emotions and ultimately achieves the goal of alleviating job burnout. Second, the mediating effect of relaxation experience between physical activity and nurses’ job burnout is established, which verifies Hypothesis H2b. Relaxation experience is a passive recovery experience, that is, a psychological experience associated with low sympathetic nerve activation (40). This low - activation state can block the resource loss of workers. Therefore, when workers feel relaxed during non - working hours, they will no longer continuously consume resources due to work requirements, thereby alleviating tension and stress and effectively repairing the resource level (69). Studies have shown that participating in physical activity during non - working hours helps improve individuals’ level of relaxation experience (68). In addition, meta - analysis also found that relaxation experience is positively correlated with high - energy levels during working hours (64). Therefore, physical activity can help nurses obtain relaxation experience during non - working hours and then alleviate job burnout. This finding not only supports the theory of resource conservation but also provides new intervention ideas for improving nurses’ mental health. Hospital managers can optimize the physical activity environment and formulate exercise plans adapted to the shift system to help nurses reduce job burnout by improving their level of relaxation experience. Third, the mediating effect of mastery experience between physical activity and nurses’ job burnout is established, which verifies H2c. Mastery experience refers to the psychological experience obtained by overcoming new challenges unrelated to work or learning new skills (40). Different from relaxation experience, it is an active recovery experience. Participating in physical activity can make workers feel their own abilities and attractiveness (35), thereby bringing a higher level of mastery experience (70). When nurses participate in physical activity after work, especially in sports such as badminton and table tennis, they are often full of challenges throughout the exercise process. Although the process of facing challenges and learning new skills inevitably involves resource consumption, physical activity provides nurses with a controllable environment. They can freely choose which type of physical activity to participate in and can also basically self - control the time they spend on these physical activities. Nurses need to face emergencies and high - emotional labor in their daily work and have been in a low - control state for a long time (71). Participating in physical activities makes up for the lack of autonomy among nurses in their work (72). Therefore, physical activity can alleviate job burnout by enhancing nurses’ mastery experience and improving their adaptability to the external environment. This finding further reveals that physical activity is not only a way to recover physical and mental resources but also helps nurses build a stronger sense of self - control and enhance their confidence in facing work challenges. In conclusion, through in - depth exploration of the relationship between physical activity and nurses’ job burnout and its internal mechanisms, this study reveals multiple mitigation mechanisms of physical activity for the job burnout of nursing staff. Specifically, the study confirms that physical activity reduces job burnout through three paths: promoting psychological disengagement, relaxation experience, and mastery experience.

Fourth, the mediating role of control experience in the relationship between physical activity and nurse burnout was not supported, and Hypothesis H2d was not confirmed. Specifically, the path from control experience to job burnout was not statistically significant. According to the conservation of resources theory, individuals are likely to experience burnout when job demands exceed their personal and job-related resources (73). In this context, for nurses—a professional group characterized by high pressure, high responsibility, and low autonomy (74)—long-term exposure to low-control work environments may diminish the psychological restorative power of short-term control experiences (75). Moreover, control experience lacks emotional arousal and direct positive feedback mechanisms, making it less effective than psychological detachment, relaxation, or mastery experiences in regulating stress responses or restoring positive emotional states. Therefore, even if nurses gain a certain sense of control through physical activity, this psychological resource may be insufficient to counteract the prolonged stress inherent in their work. In other words, there is a lack of direct correspondence between control experience and the core resources depleted by job burnout, resulting in its non-significant mediating role. This finding suggests that, although physical activity can enhance an individual’s sense of control, this psychological resource does not play a substantial role in alleviating job burnout. It indicates that control experience may serve only as a peripheral and supportive resource in this pathway, rather than a core restorative mechanism for mitigating nurse burnout.

Furthermore, although physical exercise may help alleviate nurses’ job burnout, its restorative benefits can be constrained by various subjective and objective factors. One key subjective factor is exercise motivation. Previous studies have found that the restorative effect of physical activity is maximized only when employees are intrinsically motivated to engage in exercise; when driven primarily by external pressures, the benefits are limited (34). Additionally, objective constraints such as irregular shift schedules (76), heavy workloads, and limited access to exercise facilities (77) can reduce both the frequency and quality of physical activity. These constraints may weaken the “resource replenishment” process proposed by the Conservation of Resources Theory, thereby diminishing the restorative effect of exercise on job burnout. Therefore, future interventions should not only encourage nurses to participate in physical activity but also focus on fostering intrinsic motivation and reducing environmental barriers—for example, by providing flexible scheduling and convenient exercise facilities—to maximize the restorative benefits of physical activity.

This finding indicates that recovery experiences play an important mediating role between physical activity and nurses’ job burnout and also answers the important question of why physical activity can alleviate job burnout. The findings of this study suggest that hospital administrators should consider integrating physical exercise into occupational health promotion programs. For example, self-liberation interventions, such as distributing pedometers combined with behavioral commitment strategies, have been shown to effectively encourage physical activity among sedentary nursing staff (78), thereby creating more convenient conditions for nurses to engage in physical exercise. Additionally, integrating physical activity with professional development could be explored—for instance, incorporating training modules that combine clinical skills with physical fitness exercises (e.g., physical training in simulated emergency scenarios). This approach allows exercise to serve not only as an outlet for stress relief but also as a means to enhance occupational competence. Based on these insights, the present study elucidates the pathway of “physical activity—psychological resource regeneration—alleviation of job burnout,” providing both theoretical foundations and practical guidance for healthcare institutions seeking to mitigate nurses’ job burnout. It should be emphasized that this study is based on cross-sectional correlational data and therefore cannot establish causal relationships; the related recommendations should be further tested through longitudinal or intervention studies.

Notably, future research should further explore the roles of psychological resilience and lifestyle as potential factors. Psychological resilience is regarded as a crucial psychological resource enabling individuals to cope with complex challenges and sustained stress in the workplace (79) and has been identified as an important factor in alleviating nurse burnout (42). This perspective offers new directions for expanding theoretical models. Meanwhile, lifestyle constitutes a significant contextual factor influencing individual recovery and job burnout, with positive lifestyles showing significant associations with occupational health (80). Therefore, incorporating psychological resilience and lifestyle into future theoretical frameworks will not only help reveal a more comprehensive mechanism underlying the relationship between physical activity and job burnout but also provide stronger theoretical support and practical guidance for developing integrated intervention strategies.

## 7 Conclusion

Based on the theory of conservation of resources, this study examined the relationship between physical activity and nurses’ job burnout and tested the mediating role of each dimension of recovery experiences in this association. The results indicated that physical activity was significantly and negatively associated with nurses’ job burnout, and greater participation in physical activity was correlated with higher levels of recovery experiences. Psychological disengagement, relaxation experience, and mastery experience were found to mediate the association between physical activity and nurses’ job burnout, whereas the mediating effect of control experience was not supported.

## 8 Research limitations and prospects

Although this study provides an initial exploration of the relationship between physical exercise and job burnout among nurses, several limitations should be acknowledged.

First, from a research design perspective, the study employed a cross-sectional design, which is effective for identifying associations between variables and exploring potential underlying mechanisms. However, because data were collected at a single point in time, the temporal order among variables cannot be established, limiting the ability to draw direct causal inferences. To more comprehensively and accurately verify causal relationships, future research could adopt longitudinal designs, collecting data at multiple time points to dynamically observe changes in variables and their mutual influences, thereby providing stronger support for causal inference and theoretical development.

Second, the Physical Activity Rating Scale used in this study is a brief self-report instrument. While it has demonstrated good reliability and validity in the Chinese context and is convenient for participants to complete, it may be affected by recall bias and social desirability effects. Moreover, its relatively outdated scoring formula may not adequately capture differences across various dimensions of physical activity. In addition, all variables in this study were measured using self-report questionnaires at a single time point, which may introduce common method bias. Future studies could integrate more refined and internationally standardized measurement tools or wearable devices, and incorporate multi-source, multi-wave data collection to enhance measurement accuracy and the robustness of conclusions.

Third, this study used convenience sampling and recruited nurses from three provinces in China, which limits the representativeness of the sample and may affect the external validity of the findings. Future research could employ stratified random sampling or multi-stage sampling across a broader range of regions and cultural contexts to ensure a more balanced and representative sample structure, thereby improving external validity and generalizability.

Fourth, although this study found that physical exercise has a positive effect on alleviating nurses’ job burnout, this beneficial recovery effect may be constrained by various subjective and objective factors. For example, prior research has shown that employees’ motivation for exercise influences the recovery benefits of physical activity—only when exercise is driven by intrinsic motivation does it significantly relate to recovery levels. These factors may moderate the recovery effect of physical activity, and future research should further examine their underlying mechanisms.

Finally, although this study controlled for basic demographic variables such as gender, educational attainment, marital status, and department type, it did not include work-related contextual factors closely associated with job burnout—such as workload, organizational support, and shift frequency—which may have limited the explanatory power of the model. Future research could incorporate a more comprehensive set of organizational context variables to gain deeper insights into the mechanisms linking physical exercise and nurses’ job burnout.

## Data Availability

The raw data supporting the conclusions of this article will be made available by the authors, without undue reservation.
